# Intraductal Papillary Neoplasm of the Bile Duct: A Rare Case of Intrahepatic Space-Occupying Lesion

**DOI:** 10.7759/cureus.13063

**Published:** 2021-02-01

**Authors:** Souradeep Dutta, Praveen Upadhyay, Ankit Jain, Rajesh Nachiappa Ganesh, Vishnu Prasad Nelamangala Ramakrishnaiah

**Affiliations:** 1 Surgery, Jawaharlal Institute of Postgraduate Medical Education and Research, Puducherry, IND; 2 Pathology, Jawaharlal Institute of Postgraduate Medical Education and Research, Puducherry, IND

**Keywords:** intraductal papillary neoplasm of bile duct, ipnb, cholangiocarcinoma, bt-ipmn, biliary papillomatosis

## Abstract

Intraductal papillary neoplasm of the bile duct (IPNB) is a rare tumor and is considered one of the precursor lesions for cholangiocarcinoma. Though relatively common in the far east countries, it is uncommon in the Indian population. A 67-year-old gentleman presented with vague upper abdominal pain with no history of fever, jaundice, melena, or hematemesis. An abdominal ultrasound showed a solid cystic lesion in the left lobe of the liver with upstream dilatation of bile ducts. Computed tomography and magnetic resonance imaging showed similar findings. With a differential diagnosis of intrahepatic cholangiocarcinoma, intraductal papillary neoplasm, and biliary cystadenoma, he underwent robotic-assisted left hepatectomy. Histopathology was suggestive of IPNB. Following surgery, he had an uneventful recovery and was advised for follow-up visits every six months. A clinical, radiological, and pathological profile of this rare tumor has been described here with a review of the existing literature.

## Introduction

Intraductal papillary neoplasm of the bile duct (IPNB) is a rare bile duct epithelial tumor, accounting for 10-15% of bile duct tumors [1]. IPNB is considered as the biliary counterpart of the intraductal mucinous papillary neoplasm of the pancreas [2]. It predominantly has a papillary growth pattern with a tendency to progress into invasive cholangiocarcinoma. Reported mostly from far east countries, IPNB is a rare entity among the Indian population [3,4]. Here, we report a case of IPNB in a 67-year-old male from South India who underwent a left hepatectomy.

## Case presentation

**Figure 1:**
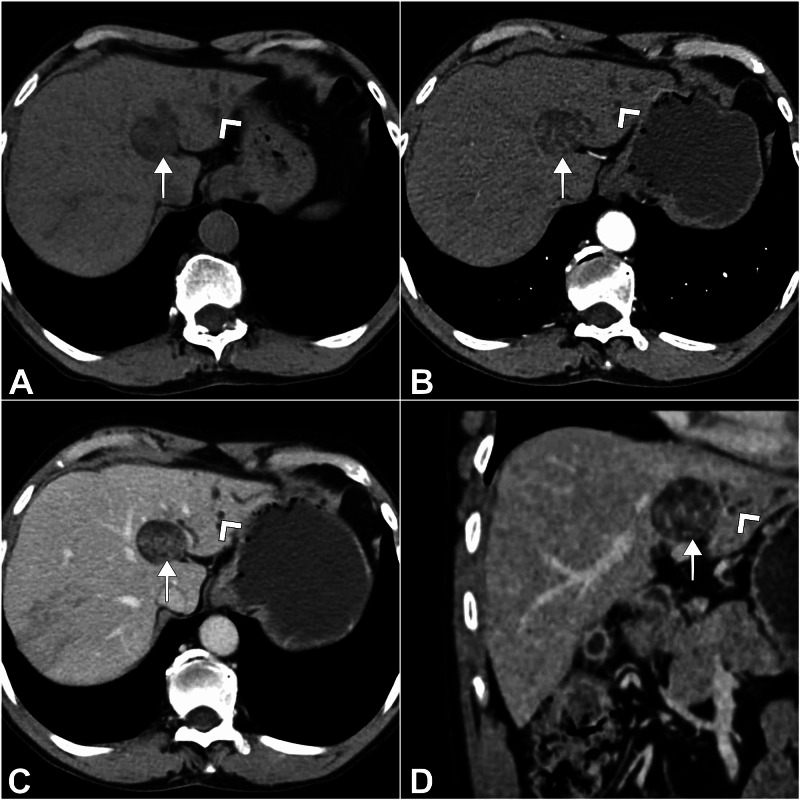
CT images. IPNB lesion is marked with a white arrow. Dilated left-sided biliary radicles are shown with white arrowheads. (A) Axial section, non-contrast (plain) phase; (B) axial section, arterial phase; (C) axial section, portal phase; (D) reconstructed coronal section, delayed phase. CT, computed tomography; IPNB, intraductal papillary neoplasm of the bile duct

A 67-year-old male from Chennai, India, presented with intermittent vague right hypochondriac pain for one month. There was no history of fever, jaundice, melena, or hematemesis. General physical and abdominal examinations were unremarkable. He self-prescribed an abdominal ultrasound at a local clinic, where he was told to have a liver tumor. His blood workup and tumor markers (carcinoembryonic antigen and alpha fetoprotein) were within normal limits. Ultrasound showed a 3 × 4-cm well-defined hypoechoic lesion with central echogenicity in segments 4a and 2 of the liver, with upstream dilatation of biliary radicles in the left medial segment. Contrast-enhanced computed tomography (CECT) showed a solid cystic lesion in segments 4a and 2 with proximal dilatation of left hepatic ducts. There was a persistent delayed intra-lesional enhancement on the post-contrast study with atrophy and volume loss of the left lobe of the liver (Figure 1).

**Figure 2:**
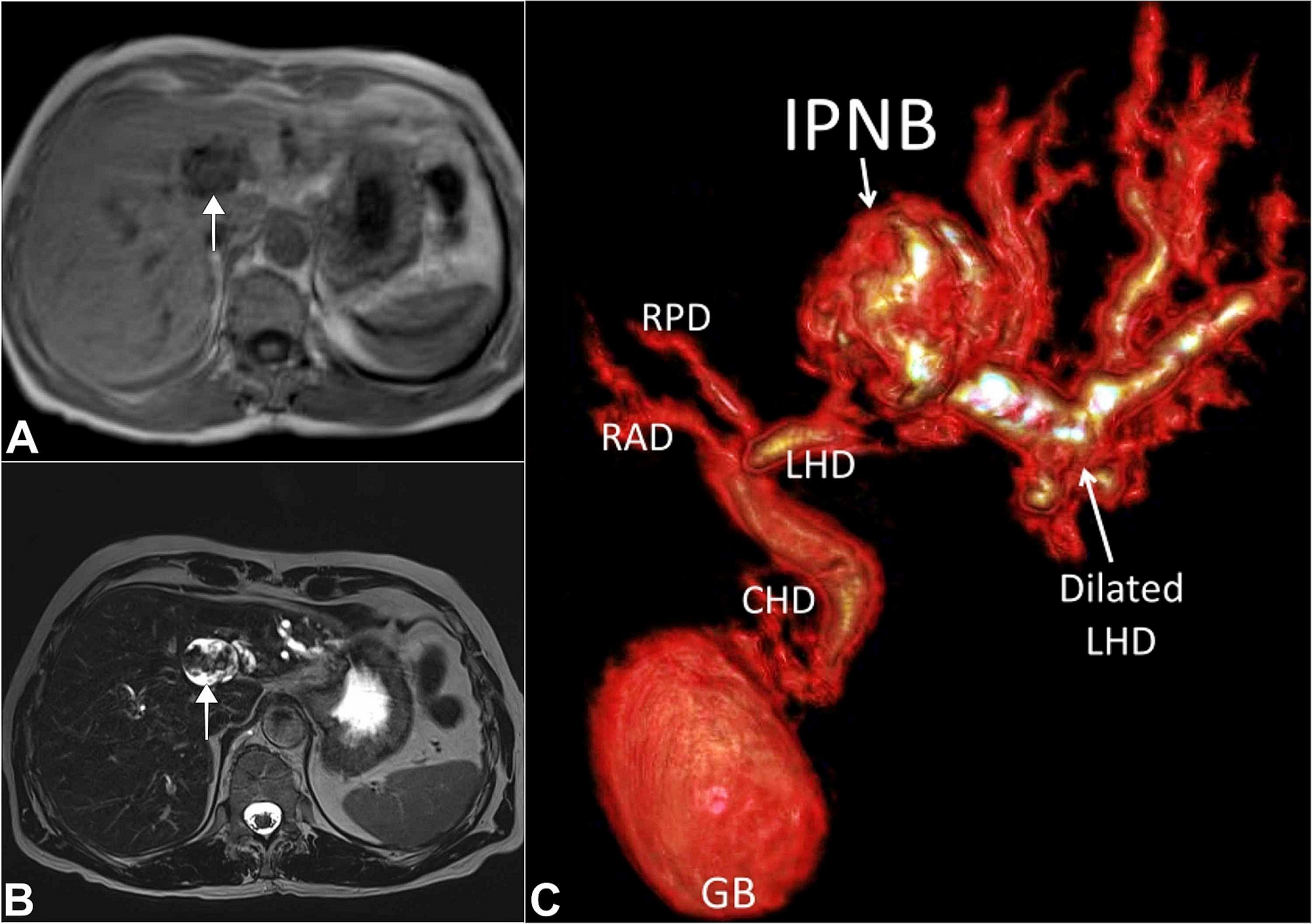
MRI images. (A) T1-weighted axial section with low to isointense lesion (white arrow); (B) T2-weighted axial section with hyperintense lesion with intraductal papillary projections (white arrow); (C) 3D volume reconstructed MRCP image showing the cystic lesion with communication with LHD which is causing proximal dilatations of the biliary ducts. MRI, magnetic resonance imaging; MRCP, magnetic resonance cholangiopancreatography; RAD, right anterior sectoral duct; RPD, right posterior sectoral duct; GB, gall bladder; LHD, left hepatic duct

Magnetic resonance imaging (MRI) showed a well-defined oval-shaped heterogenous lesion having an intraductal communication with left-sided biliary radicles (Figure 2).

**Figure 3:**
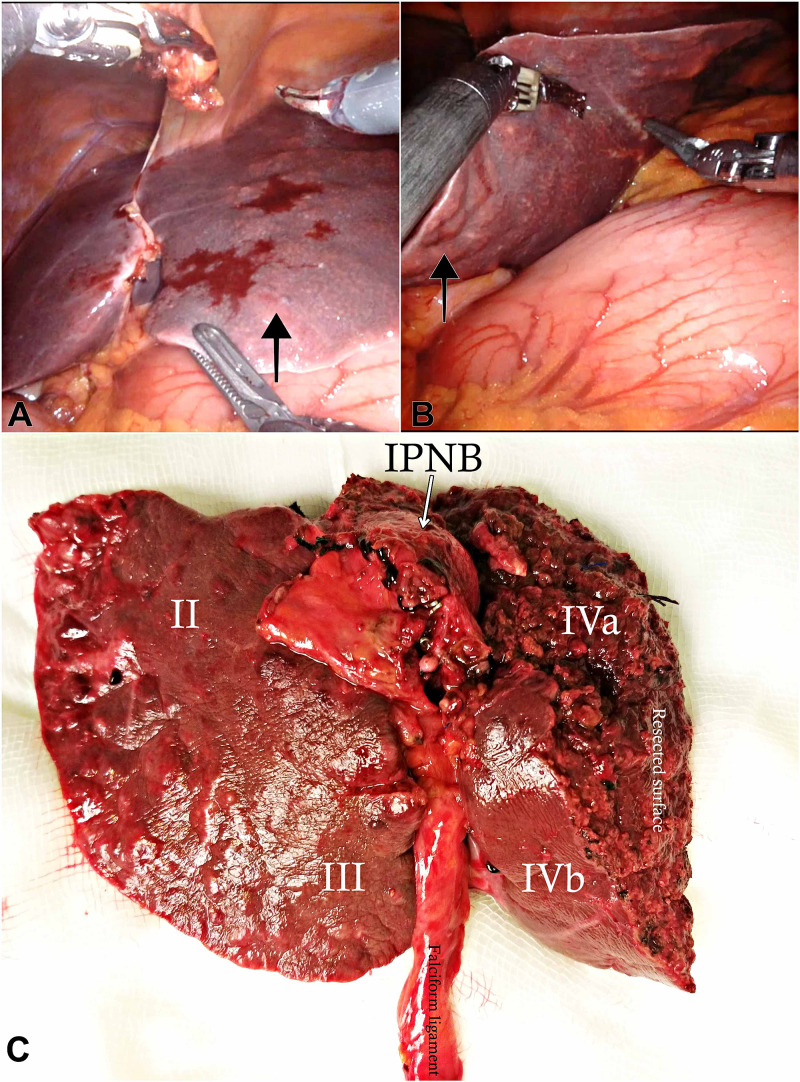
(A, B) Intraoperative robotic surgery images showing the nodular surface of the left lobe of the liver (black arrows) in contrast to the smooth surface of the right side; (C) resected specimen of the left lobe of the liver (posterior surface). II, III, IVa, IVb, demonstrating the respective liver segments; IPNB, intraductal papillary neoplasm of the bile duct

Based on the above findings, with differentials of intrahepatic cholangiocarcinoma, intraductal papillary neoplasm, or biliary cystadenoma, the patient underwent a robotic-assisted left hepatectomy. The left hepatic duct was resected at its origin, keeping the right hepatic ducts and gall bladder intact (Figure 3).

**Figure 4:**
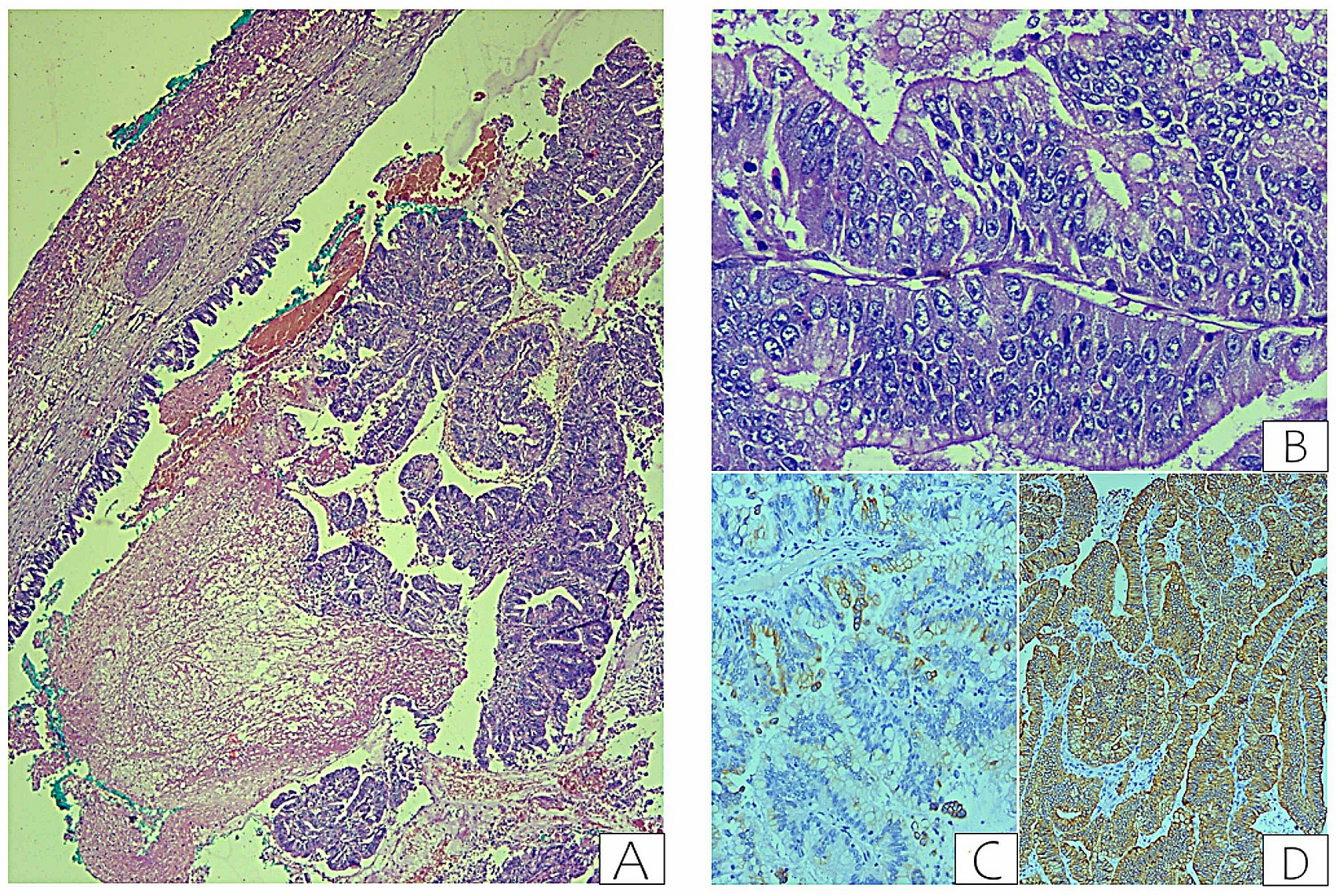
Histopathological images. (A) Section showing a cystic lesion with a thin rim of liver parenchyma (painted end). The cyst is lined by columnar epithelium thrown into complex papillary processes, hematoxylin and eosin stain, ×40; (B) Section showing higher magnification of the lesion with stratified lining exhibiting prominent nucleoli, occasional mitoses, and moderate nuclear pleomorphism with evidence of intracytoplasmic mucin, hematoxylin and eosin stain, ×400; (C) Section showing tumor cells exhibiting spare cytoplasmic expression of cytokeratin 7. Immunohistochemistry with DAKO polyclonal antibody for cytokeratin 7, diaminobenzidine stain, ×200; (D) Section showing tumor cells exhibiting strong diffuse cytoplasmic expression of cytokeratin 19. Immunohistochemistry with DAKO polyclonal antibody for cytokeratin 19, diaminobenzidine stain, ×200.

Gross examination of the specimen revealed a cystic lesion of size 3 × 3 × 2.5 cm filled with friable papillary excrescences, occupying the entire cavity with multiple upstream bile ducts’ dilatation. A microscopic examination from the cystic lesion showed a dilated bile duct with a papillary structure with a fibrovascular core. Ovarian stroma was not seen with no evidence of invasive stromal carcinoma. On immunohistochemistry, cells were positive for cytokeratin (CK) 19 and focally positive for CK 7 (Figure 4).

The above findings were consistent with that of IPNB. The patient stayed in the intensive care unit for a day, following which he was transferred to the general surgical ward, where the recovery was uneventful. Oral feeds were started on day three, and he was discharged on the eighth postoperative day. He was discharged with the advice of six-monthly follow-up.

## Discussion

IPNB is a rare tumor initially described in 1976 as multicentric biliary papillomatosis associated with invasive adenocarcinoma [5]. The latest 2010 World Health Organization (WHO) classifications define IPNB as a “papillary or villous neoplasm covering delicate fibrovascular stalks occurring in the bile ducts” [6]. WHO further classifies IPNB as IPN with low- or intermediate-grade intraepithelial neoplasia, IPN with high-grade intraepithelial neoplasia, and IPN with an associated invasive carcinoma. Most cases of IPNB are reported from far eastern countries like Japan, Taiwan, China, and Korea [5]. Higher incidence in these countries can be attributed to a higher prevalence of risk factors such as endemic hepatolithiasis and Clonorchis infection [7]. Other risk factors include primary sclerosing cholangitis, choledochal cyst, familial adenomatous polyposis, or Gardner syndrome [8].

In most series, the median age of IPNB is 60-66 years, with a male predominance [1,9]. Patients with IPNB present with right hypochondriac pain (most common, 35-88%), cholangitis, or obstructive jaundice. However, some patients can be completely asymptomatic, being diagnosed incidentally. Radiologically, ultrasound may show intermediate echogenic mass within a dilated duct. Proximal duct dilatation is one of the characteristic features, with an occasional finding of focal liver atrophy. On CECT, IPNB can have various features, including intrahepatic and extrahepatic duct dilatation, intraductal mass, which can be iso- or hyper-enhancing compared to the adjacent liver with enhancement in the late arterial phase. MRI can show solid lesion, low or isointense in T1, and slightly hyperintense in T2-weighted sequences. In MRCP, direct visualization of the papillary lesion may be possible. It typically involves the hilum or the intrahepatic ducts. When intrahepatic, the involvement of left-sided ducts is most common [10].

Histologically, IPNB can be subdivided into four major types: pancreatobiliary, intestinal, gastric, and oncocytic [11]. The majority of pancreatobilliary type express MUC1 and CK-7, and very few of them are positive for CDX2, the gastric subtype is positive for MUC1 and CK7 and negative for CDX2, the intestinal type is positive for both CK 7 and CK 20, and oncocytic type is positive for MU5AC and MU6. Our case is of pancreatobilliary type with focal and weakly positive for CK 7.

Even though all IPNB lesions are not malignant, they can often cause obstructive jaundice with or without recurrent cholangitis and can progress to invasive cholangiocarcinoma [9,12]. Hence, they should be considered for surgical treatment. Patients not having any evidence of distant metastasis should undergo surgical resection, which should be major hepatectomy with or without extrahepatic bile duct resection. In comparison to cholangiocarcinoma, IPNB has a better prognosis. With curative resection, the five-year survival rate is between 47% and 84%. The recurrence rate depends on the lesion’s invasiveness and is estimated to be in the range of 20% to 60% [13].

## Conclusions

Although IPNB is a rare entity among the Indian population and is difficult to diagnose preoperatively, it should be in the list of differentials of such a cystic space-occupying lesion of the liver. As IPNB is a premalignant lesion and is known to cause recurrent cholangitis and obstructive jaundice, surgical resection with free margins is always indicated. With proper surgical management, patients with IPNB can have a good long-term prognosis.
